# 3-[(*E*)-2-(2-Meth­oxy­phen­yl)vin­yl]-5,5-di­methyl­cyclo­hex-2-enone

**DOI:** 10.1107/S1600536814006527

**Published:** 2014-04-02

**Authors:** Zeenat Fatima, Govindaraj Senthilkumar, A. Vadivel, Haridoss Manikandan, Devadasan Velmurugan

**Affiliations:** a Center of Advanced Study in Crystallography and Biophysics, University of Madras, Guindy Campus, Chennai 600 025, India; b Department of Chemistry, Annamalai University, Annamalainagar 608 002, Tamilnadu, India

## Abstract

The title compound, C_17_H_20_O_2_, has an *E* conformation about the bridging C=C bond. The cyclo­hexene ring adopts an envelope conformation with the dimethyl-substituted C atom as the flap. Its mean plane makes a dihedral angle of 7.20 (12)° with the benzene ring. In the crystal, neighbouring mol­ecules are connected *via* C—H⋯O hydrogen bonds, forming chains running along the *a*-axis direction.

## Related literature   

For the pharmacological activity of cyclo­hexa­none derivatives, see: Puetz *et al.* (2003 ▶); Rajveer *et al.* (2010 ▶). For related structures, see: Fatima *et al.* (2013 ▶); Hema *et al.* (2006 ▶). For ring puckering parametes, see: Cremer & Pople (1975 ▶).
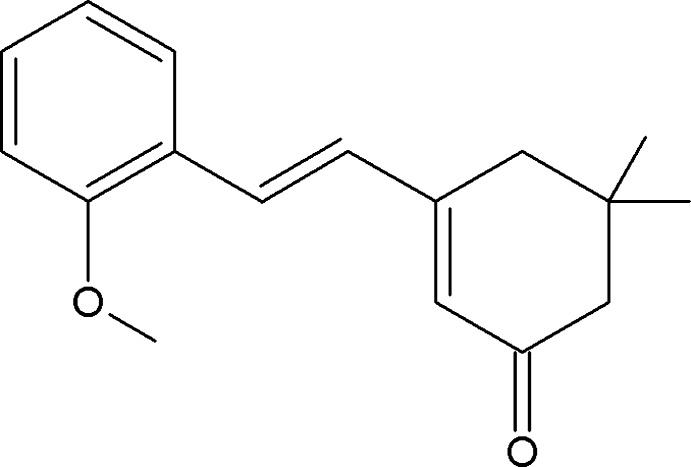


## Experimental   

### 

#### Crystal data  

C_17_H_20_O_2_
*M* *_r_* = 256.33
Monoclinic, 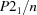

*a* = 7.208 (4) Å
*b* = 13.824 (7) Å
*c* = 15.022 (8) Å
β = 92.13 (2)°
*V* = 1495.8 (14) Å^3^
*Z* = 4
Mo *K*α radiation
μ = 0.07 mm^−1^
*T* = 293 K
0.30 × 0.25 × 0.20 mm

#### Data collection  

Bruker SMART APEXII area-detector diffractometer
Absorption correction: multi-scan (*SADABS*; Bruker, 2008 ▶) *T*_min_ = 0.978, *T*_max_ = 0.986
10491 measured reflections
3517 independent reflections
2489 reflections with *I* > 2σ(*I*)
*R*_int_ = 0.054

#### Refinement  

*R*[*F* ^2^ > 2σ(*F* ^2^)] = 0.085
*wR*(*F* ^2^) = 0.283
*S* = 1.06
3517 reflections
175 parameters
H-atom parameters constrained
Δρ_max_ = 0.65 e Å^−3^
Δρ_min_ = −0.27 e Å^−3^

### 

Data collection: *APEX2* (Bruker, 2008 ▶); cell refinement: *SAINT* (Bruker, 2008 ▶); data reduction: *SAINT*; program(s) used to solve structure: *SHELXS97* (Sheldrick, 2008 ▶); program(s) used to refine structure: *SHELXL97* (Sheldrick, 2008 ▶); molecular graphics: *ORTEP-3 for Windows* (Farrugia, 2012 ▶); software used to prepare material for publication: *SHELXL97* and *PLATON* (Spek, 2009 ▶).

## Supplementary Material

Crystal structure: contains datablock(s) global, I. DOI: 10.1107/S1600536814006527/su2717sup1.cif

Structure factors: contains datablock(s) I. DOI: 10.1107/S1600536814006527/su2717Isup2.hkl

Supporting information file. DOI: 10.1107/S1600536814006527/su2717Isup3.cml

CCDC reference: 993438

Additional supporting information:  crystallographic information; 3D view; checkCIF report

## Figures and Tables

**Table 1:** Hydrogen-bond geometry (Å, °)

*D*—H⋯*A*	*D*—H	H⋯*A*	*D*⋯*A*	*D*—H⋯*A*
C14—H14*B*⋯O1^i^	0.97	2.62	3.554 (4)	161

Symmetry code: (i) 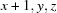.

**Fig. 1.**
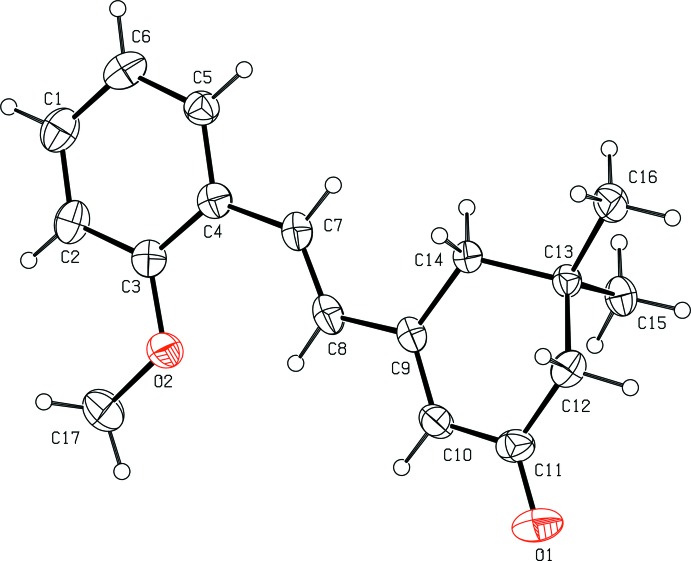
The molecular structure of the title molecule, with atom labelling. The displacement ellipsoids are drawn at the 30% probability level.

**Fig. 2.**
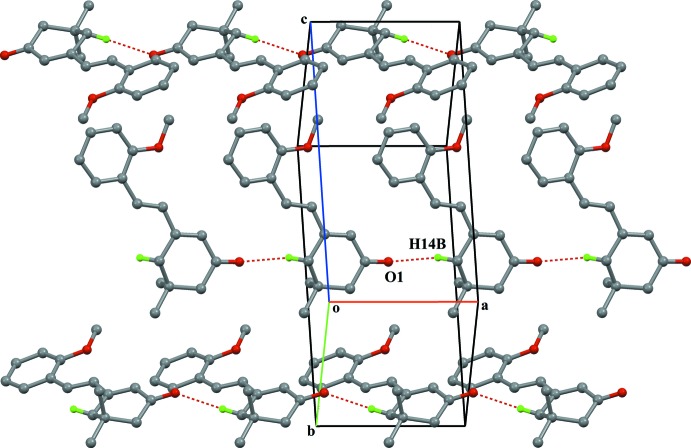
The crystal packing of the title compound viewed along the b axis. The hydrogen bonds are shown as dashed lines (see Table 1 for details; H-atoms not involved in hydrogen bonding have been excluded for clarity).

## supplementary crystallographic information

### 1. Comment

Cyclohexanone is an aliphatic cyclic ketone. Cyclohexanone derivatives have potent pharmacological activity in the treatment of a broad spectrum of medical conditions (Puetz *et al.*, 2003). The cyclohexanone moiety constitutes an important structural feature in several anti-inflammatory, analgesic, local anesthetic and antihistaminic drugs (Rajveer *et al.*, 2010). As part of our studies in this area (Fatima *et al.*, 2013; Hema *et al.*, 2006), we have undertaken a single-crystal structure determination of the title compound.

In the title compound, Fig. 1, the cyclohexene ring (C9—C14) adopts an envelope conformation with atom C13 as the flap: puckering parameters (Cremer & Pople, 1975) are Q = 0.464 (3) Å, θ = 52.3 (4) °, and φ = 232.2 (4) °. Its mean plane makes a dihedral angle of 7.20 (12)° with the benzene ring (C1—C6).

In the crystal, hydrogen bonded chains running along the *a*-axis direction are generated by connecting neighbouring molecules via C—H···O hydrogen bonds (Table 1 and Fig. 2).

### 2. Experimental

A mixture of isophorone (0.01 mol), 2-methoxybezaldehyde (0.01 mol) and sodium hydroxide solution (10 ml, 10%) in ethanol (25 ml) was stirred at room temperature until the starting material disappeared. The resulting mixture was poured into crushed ice and the precipitate was filtered off, dried and recrystallized from ethanol giving p. colourless block-like crystals [Yield = 93%; M.p. = 373-375 K].

### 3. Refinement

The H atoms were placed in calculated positions and refined as riding atoms: C—H = 0.93 - 0.97 Å with *U*_iso_(H) = 1.5*U*_eq_(C-methyl ) and = 1.2*U*_eq_(C) for other H atoms.

### Crystal data

**Table:**

C_17_H_20_O_2_	*F*(000) = 552
*M**_r_* = 256.33	*D*_x_ = 1.138 Mg m^−^^3^
Monoclinic, *P*2_1_/*n*	Mo *K*α radiation, λ = 0.71073 Å
Hall symbol: -P 2yn	Cell parameters from 3517 reflections
*a* = 7.208 (4) Å	θ = 2.0–28.6°
*b* = 13.824 (7) Å	µ = 0.07 mm^−^^1^
*c* = 15.022 (8) Å	*T* = 293 K
β = 92.13 (2)°	Block, colourless
*V* = 1495.8 (14) Å^3^	0.30 × 0.25 × 0.20 mm
*Z* = 4	

### Data collection

**Table:**

Bruker SMART APEXII area-detector diffractometer	3517 independent reflections
Radiation source: fine-focus sealed tube	2489 reflections with *I* > 2σ(*I*)
Graphite monochromator	*R*_int_ = 0.054
ω and φ scans	θ_max_ = 28.6°, θ_min_ = 2.0°
Absorption correction: multi-scan (*SADABS*; Bruker, 2008)	*h* = −9→9
*T*_min_ = 0.978, *T*_max_ = 0.986	*k* = −18→17
10491 measured reflections	*l* = −19→11

### Refinement

**Table:**

Refinement on *F*^2^	Primary atom site location: structure-invariant direct methods
Least-squares matrix: full	Secondary atom site location: difference Fourier map
*R*[*F*^2^ > 2σ(*F*^2^)] = 0.085	Hydrogen site location: inferred from neighbouring sites
*wR*(*F*^2^) = 0.283	H-atom parameters constrained
*S* = 1.06	*w* = 1/[σ^2^(*F*_o_^2^) + (0.1498*P*)^2^ + 0.8245*P*] where *P* = (*F*_o_^2^ + 2*F*_c_^2^)/3
3517 reflections	(Δ/σ)_max_ < 0.001
175 parameters	Δρ_max_ = 0.65 e Å^−^^3^
0 restraints	Δρ_min_ = −0.27 e Å^−^^3^

### Special details

**Table:**

Geometry. All e.s.d.'s (except the e.s.d. in the dihedral angle between two l.s. planes) are estimated using the full covariance matrix. The cell e.s.d.'s are taken into account individually in the estimation of e.s.d.'s in distances, angles and torsion angles; correlations between e.s.d.'s in cell parameters are only used when they are defined by crystal symmetry. An approximate (isotropic) treatment of cell e.s.d.'s is used for estimating e.s.d.'s involving l.s. planes.
Refinement. Refinement of *F*^2^ against ALL reflections. The weighted *R*-factor *wR* and goodness of fit *S* are based on *F*^2^, conventional *R*-factors *R* are based on *F*, with *F* set to zero for negative *F*^2^. The threshold expression of *F*^2^ > σ(*F*^2^) is used only for calculating *R*-factors(gt) *etc*. and is not relevant to the choice of reflections for refinement. *R*-factors based on *F*^2^ are statistically about twice as large as those based on *F*, and *R*- factors based on ALL data will be even larger.

### Fractional atomic coordinates and isotropic or equivalent isotropic displacement parameters (Å^2^)

**Table:**

	*x*	*y*	*z*	*U*_iso_*/*U*_eq_	
C1	1.4696 (5)	0.6141 (2)	0.2237 (2)	0.0617 (8)	
H1	1.5376	0.6552	0.1884	0.074*	
C2	1.2899 (4)	0.5897 (2)	0.1978 (2)	0.0533 (7)	
H2	1.2377	0.6147	0.1451	0.064*	
C3	1.1846 (4)	0.52740 (19)	0.25024 (18)	0.0472 (6)	
C4	1.2616 (4)	0.49045 (18)	0.33219 (18)	0.0452 (6)	
C5	1.4433 (4)	0.51557 (18)	0.35727 (18)	0.0464 (6)	
H5	1.4951	0.4915	0.4104	0.056*	
C6	1.5515 (4)	0.5767 (2)	0.3041 (2)	0.0617 (8)	
H6	1.6732	0.5920	0.3213	0.074*	
C7	1.1621 (4)	0.42686 (18)	0.39463 (18)	0.0479 (6)	
H7	1.2321	0.4088	0.4452	0.057*	
C8	0.9882 (4)	0.39088 (18)	0.39058 (18)	0.0500 (7)	
H8	0.9143	0.4049	0.3401	0.060*	
C9	0.9055 (3)	0.33023 (16)	0.46110 (16)	0.0411 (6)	
C10	0.7259 (4)	0.29964 (19)	0.45218 (19)	0.0496 (7)	
H10	0.6585	0.3153	0.4002	0.060*	
C11	0.6334 (3)	0.24317 (19)	0.5206 (2)	0.0499 (7)	
C12	0.7438 (4)	0.22367 (19)	0.60826 (18)	0.0459 (6)	
H12A	0.6987	0.1644	0.6345	0.055*	
H12B	0.7219	0.2760	0.6495	0.055*	
C13	0.9558 (3)	0.21429 (15)	0.59556 (15)	0.0369 (5)	
C14	1.0219 (3)	0.30514 (16)	0.54545 (16)	0.0396 (5)	
H14A	1.0200	0.3600	0.5857	0.048*	
H14B	1.1495	0.2952	0.5292	0.048*	
C15	0.9938 (4)	0.12099 (17)	0.54174 (19)	0.0480 (6)	
H15A	0.9532	0.0657	0.5744	0.072*	
H15B	1.1244	0.1157	0.5323	0.072*	
H15C	0.9272	0.1239	0.4853	0.072*	
C16	1.0603 (5)	0.2081 (2)	0.68771 (18)	0.0563 (8)	
H16A	1.0354	0.2652	0.7216	0.084*	
H16B	1.1913	0.2031	0.6793	0.084*	
H16C	1.0187	0.1521	0.7192	0.084*	
C17	0.9273 (5)	0.5303 (3)	0.1418 (2)	0.0736 (10)	
H17A	0.9271	0.5997	0.1389	0.110*	
H17B	0.8023	0.5068	0.1344	0.110*	
H17C	1.0008	0.5048	0.0952	0.110*	
O1	0.4730 (3)	0.2135 (2)	0.5081 (2)	0.0762 (8)	
O2	1.0044 (3)	0.49973 (15)	0.22679 (13)	0.0569 (6)	

### Atomic displacement parameters (Å^2^)

**Table:**

	*U*^11^	*U*^22^	*U*^33^	*U*^12^	*U*^13^	*U*^23^
C1	0.0701 (18)	0.0601 (16)	0.0560 (17)	−0.0103 (15)	0.0175 (15)	−0.0004 (14)
C2	0.0659 (16)	0.0489 (13)	0.0464 (14)	−0.0038 (12)	0.0184 (13)	−0.0005 (11)
C3	0.0542 (14)	0.0450 (12)	0.0426 (13)	0.0002 (11)	0.0037 (12)	−0.0020 (10)
C4	0.0477 (13)	0.0433 (12)	0.0448 (13)	0.0021 (10)	0.0033 (11)	−0.0035 (10)
C5	0.0477 (13)	0.0468 (12)	0.0445 (13)	0.0005 (10)	−0.0014 (11)	−0.0020 (10)
C6	0.0517 (14)	0.0652 (17)	0.069 (2)	−0.0135 (13)	0.0093 (15)	−0.0085 (15)
C7	0.0576 (14)	0.0416 (12)	0.0446 (13)	0.0017 (11)	0.0024 (12)	0.0073 (10)
C8	0.0646 (16)	0.0446 (12)	0.0404 (13)	0.0084 (12)	−0.0033 (12)	0.0079 (10)
C9	0.0488 (12)	0.0367 (10)	0.0375 (12)	0.0056 (9)	−0.0005 (10)	0.0036 (9)
C10	0.0490 (13)	0.0494 (13)	0.0495 (14)	0.0067 (11)	−0.0090 (12)	0.0045 (11)
C11	0.0390 (12)	0.0479 (13)	0.0627 (17)	0.0036 (10)	0.0002 (12)	−0.0098 (12)
C12	0.0479 (13)	0.0456 (12)	0.0448 (13)	−0.0035 (10)	0.0127 (11)	−0.0038 (10)
C13	0.0432 (11)	0.0346 (10)	0.0327 (11)	−0.0005 (9)	0.0005 (9)	0.0022 (8)
C14	0.0409 (11)	0.0376 (11)	0.0401 (12)	−0.0020 (9)	−0.0014 (10)	0.0062 (9)
C15	0.0581 (14)	0.0363 (11)	0.0497 (14)	0.0092 (10)	0.0051 (12)	−0.0005 (10)
C16	0.0710 (18)	0.0579 (15)	0.0392 (13)	−0.0071 (13)	−0.0096 (13)	0.0101 (11)
C17	0.084 (2)	0.083 (2)	0.0520 (18)	−0.0015 (19)	−0.0171 (17)	0.0147 (16)
O1	0.0395 (10)	0.0955 (18)	0.0935 (19)	−0.0095 (11)	−0.0002 (11)	−0.0084 (14)
O2	0.0559 (11)	0.0680 (12)	0.0461 (11)	−0.0088 (9)	−0.0081 (9)	0.0163 (9)

### Geometric parameters (Å, º)

**Table:**

C1—C2	1.381 (4)	C11—O1	1.234 (3)
C1—C6	1.421 (5)	C11—C12	1.537 (4)
C1—H1	0.9300	C12—C13	1.553 (4)
C2—C3	1.408 (4)	C12—H12A	0.9700
C2—H2	0.9300	C12—H12B	0.9700
C3—O2	1.387 (3)	C13—C14	1.548 (3)
C3—C4	1.426 (4)	C13—C15	1.552 (3)
C4—C5	1.393 (4)	C13—C16	1.553 (3)
C4—C7	1.489 (4)	C14—H14A	0.9700
C5—C6	1.416 (4)	C14—H14B	0.9700
C5—H5	0.9300	C15—H15A	0.9600
C6—H6	0.9300	C15—H15B	0.9600
C7—C8	1.348 (4)	C15—H15C	0.9600
C7—H7	0.9300	C16—H16A	0.9600
C8—C9	1.493 (4)	C16—H16B	0.9600
C8—H8	0.9300	C16—H16C	0.9600
C9—C10	1.364 (4)	C17—O2	1.437 (3)
C9—C14	1.533 (3)	C17—H17A	0.9600
C10—C11	1.470 (4)	C17—H17B	0.9600
C10—H10	0.9300	C17—H17C	0.9600
			
C2—C1—C6	120.4 (3)	C13—C12—H12A	109.0
C2—C1—H1	119.8	C11—C12—H12B	109.0
C6—C1—H1	119.8	C13—C12—H12B	109.0
C1—C2—C3	120.7 (3)	H12A—C12—H12B	107.8
C1—C2—H2	119.7	C14—C13—C15	111.0 (2)
C3—C2—H2	119.7	C14—C13—C12	108.29 (18)
O2—C3—C2	123.0 (2)	C15—C13—C12	109.07 (19)
O2—C3—C4	116.8 (2)	C14—C13—C16	109.30 (19)
C2—C3—C4	120.1 (3)	C15—C13—C16	109.2 (2)
C5—C4—C3	118.4 (3)	C12—C13—C16	110.0 (2)
C5—C4—C7	116.5 (2)	C9—C14—C13	114.63 (19)
C3—C4—C7	125.0 (2)	C9—C14—H14A	108.6
C4—C5—C6	121.8 (3)	C13—C14—H14A	108.6
C4—C5—H5	119.1	C9—C14—H14B	108.6
C6—C5—H5	119.1	C13—C14—H14B	108.6
C5—C6—C1	118.5 (3)	H14A—C14—H14B	107.6
C5—C6—H6	120.8	C13—C15—H15A	109.5
C1—C6—H6	120.8	C13—C15—H15B	109.5
C8—C7—C4	131.2 (2)	H15A—C15—H15B	109.5
C8—C7—H7	114.4	C13—C15—H15C	109.5
C4—C7—H7	114.4	H15A—C15—H15C	109.5
C7—C8—C9	124.8 (2)	H15B—C15—H15C	109.5
C7—C8—H8	117.6	C13—C16—H16A	109.5
C9—C8—H8	117.6	C13—C16—H16B	109.5
C10—C9—C8	120.5 (2)	H16A—C16—H16B	109.5
C10—C9—C14	119.9 (2)	C13—C16—H16C	109.5
C8—C9—C14	119.5 (2)	H16A—C16—H16C	109.5
C9—C10—C11	123.3 (2)	H16B—C16—H16C	109.5
C9—C10—H10	118.3	O2—C17—H17A	109.5
C11—C10—H10	118.3	O2—C17—H17B	109.5
O1—C11—C10	121.1 (3)	H17A—C17—H17B	109.5
O1—C11—C12	121.6 (3)	O2—C17—H17C	109.5
C10—C11—C12	117.3 (2)	H17A—C17—H17C	109.5
C11—C12—C13	113.0 (2)	H17B—C17—H17C	109.5
C11—C12—H12A	109.0	C3—O2—C17	118.2 (2)
			
C6—C1—C2—C3	−0.1 (5)	C8—C9—C10—C11	−177.4 (2)
C1—C2—C3—O2	−179.2 (3)	C14—C9—C10—C11	0.6 (4)
C1—C2—C3—C4	1.4 (4)	C9—C10—C11—O1	−176.8 (3)
O2—C3—C4—C5	179.1 (2)	C9—C10—C11—C12	3.7 (4)
C2—C3—C4—C5	−1.6 (4)	O1—C11—C12—C13	148.6 (3)
O2—C3—C4—C7	−1.5 (4)	C10—C11—C12—C13	−31.9 (3)
C2—C3—C4—C7	177.9 (3)	C11—C12—C13—C14	53.3 (3)
C3—C4—C5—C6	0.4 (4)	C11—C12—C13—C15	−67.5 (3)
C7—C4—C5—C6	−179.2 (3)	C11—C12—C13—C16	172.7 (2)
C4—C5—C6—C1	1.0 (4)	C10—C9—C14—C13	24.0 (3)
C2—C1—C6—C5	−1.1 (5)	C8—C9—C14—C13	−158.0 (2)
C5—C4—C7—C8	179.3 (3)	C15—C13—C14—C9	70.0 (3)
C3—C4—C7—C8	−0.2 (5)	C12—C13—C14—C9	−49.6 (3)
C4—C7—C8—C9	−177.6 (3)	C16—C13—C14—C9	−169.5 (2)
C7—C8—C9—C10	177.9 (3)	C2—C3—O2—C17	4.8 (4)
C7—C8—C9—C14	0.0 (4)	C4—C3—O2—C17	−175.8 (3)

### Hydrogen-bond geometry (Å, º)

**Table:**

*D*—H···*A*	*D*—H	H···*A*	*D*···*A*	*D*—H···*A*
C14—H14*B*···O1^i^	0.97	2.62	3.554 (4)	161

Symmetry code: (i) *x*+1, *y*, *z*.
